# Efficacy and Safety of Repeated Radiofrequency Thermocoagulation on Trigeminal Neuralgia Patients

**DOI:** 10.1111/papr.70148

**Published:** 2026-03-13

**Authors:** Antti J. Luikku, Sara Matikka, Sami Heikkinen, Anssi Lipponen, Katja Luostarinen, Timo Koivisto, Ville Leinonen, Jukka Huttunen, Henna‐Kaisa Jyrkkänen

**Affiliations:** ^1^ Neurosurgery of NeuroCenter Kuopio University Hospital Kuopio Finland; ^2^ Institute of Clinical Medicine University of Eastern Finland Kuopio Finland; ^3^ Institute of Biomedicine University of Eastern Finland Kuopio Finland; ^4^ Pain Clinic, Neurology of NeuroCenter Kuopio University Hospital Kuopio Finland

**Keywords:** complications, radiofrequency thermocoagulation, treatment response, trigeminal neuralgia

## Abstract

**Background:**

Trigeminal neuralgia (TN) is a pain condition characterized by paroxysmal, electric shock‐like facial pain, affecting one or more areas of the branches. Approximately 33%–50% of patients require invasive treatment. Radiofrequency thermocoagulation (RFTC) is an established method for managing drug‐resistant and chronic TN. This study evaluates treatment response quality and complication rates in repeated RFTC procedures for TN patients.

**Methods:**

In this retrospective analysis, patient records were extracted from the electronic medical records of Kuopio University Hospital, using the trigeminal neuralgia diagnosis code and the procedure code for thermal destruction of a cranial nerve. Data collected included sex, age, treatment outcomes at 3‐month follow‐up, presence of complications, technical details, and procedural success for each intervention.

**Results:**

Data from 140 patients were analyzed. An excellent or good response was observed in 79% of patients after the first procedure, 62.9% after the second, and 42.3% after the third. Complication rates were 15.7%, 19.6%, and 42.9%, respectively. Logistic regression analysis showed that complication risk was significantly associated with tertiary procedure and female sex. Development of *painful post‐traumatic trigeminal neuropathy* (PTTN) was more common after repeated interventions; 1.4% after the first, 3.1% after the second, and 13.8% after the third procedure.

**Conclusions:**

RFTC is an effective and safe method for treatment for persistent trigeminal neuralgia when conservative treatment fails. However, its benefits diminish, and risks increase with each additional procedure, particularly the third. Based on these findings, reintervention should generally be limited to a single repeat procedure.

## 1 Background

Trigeminal neuralgia (TN) is a severe pain disorder characterized by paroxysmal, electric shock‐like facial pain condition triggered by harmless stimuli such as light touch, eating or talking. Attacks last from seconds to minutes, and their frequency ranges from a few episodes to hundreds per day, with alternating relapses and remissions being typical [1, 2]. Approximately 14%–50% of patients also experience constant background burning or throbbing pain, the pathophysiology of which remains unclear [2, 3]. According to the International Headache Society (IHS) ICHD‐3 criteria, this subtype is classified as *Classical trigeminal Neuralgia with concomitant persistent facial pain* (previously termed *atypical trigeminus neuralgia* and *trigeminus neuralgia type‐2*) [4]. This persistent subtype responds less favorably to both conservative treatment and neurosurgical interventions [5, 6]. Etiologically, TN is categorized into classical, idiopathic, and secondary forms. Classical TN, which accounts for 80%–90% of cases, involves vascular compression of the trigeminal nerve root [1, 2, 7]. Secondary TN results from another disorder, or damage of the trigeminal nerve [4]. Idiopathic TN is a diagnosis of exclusion when no identifiable cause is found [8].

Pharmacological treatment is the first‐line approach, whereas invasive procedures are considered when medication fails or is poorly tolerated. Approximately 33%–50% of patients require surgical intervention [2]. For MRI‐negative patients or those unsuitable for open surgery, invasive ablative procedures and stereotactic radiosurgery are options [9]. Ablative techniques include radiofrequency thermocoagulation (RFTC), performed using conventional, pulsed, or combined protocols, mechanical balloon compression, or chemical neurolysis with glycerol injection [5, 8, 10].

The principle of ablative treatment is to deliberately damage pain transmitting fibers of the trigeminal nerve ganglion (Gasserian ganglion) or its branches. Consequently, the most common complication of ablative treatments is reduced facial sensation (19%–100%). Other complications include dysesthesia (6%), reduced corneal sensation (5%), and trigeminal motor weakness such as masticatory weakness (5%) [2, 8, 10, 11]. The most feared complication is *painful post‐traumatic trigeminal neuropathy* (*PTTN*), previously termed anesthesia dolorosa [12]. PTTN is a severe, chronic, and difficult‐to‐treat pain condition following trigeminal nerve trauma, often accompanied by other signs of trigeminal nerve dysfunction (2013). Its incidence is approximately 1%–4% among patients undergoing ablative procedures [2, 12].

Based on daily clinical observations, it is suggested that treatment response efficacy declines with repeated invasive ablative procedures. Therefore, this study aimed to evaluate treatment response and complication rates in patients undergoing repeated RFTC for TN.

## 2 Materials and Methods

Kuopio University Hospital (KUH) is a tertiary care center within the wellbeing services county of North Savo and provides neurosurgical treatment for the wellbeing service counties of South Savo, Central Finland, and North Karelian. The population of catchment area is 850,000.

Data from patients treated with Gasserian Ganglion RFTC at the KUH between January 1st, 1998 and July 31st, 2024, were retrospectively analyzed. Patient records were collected based on the ICD‐10 diagnosis code G50.0 (trigeminal neuralgia) and the NOMESCO (Nordic Medico‐Statistical Committee) procedure code AAH30 (thermal destruction of cranial nerve). Records from neurosurgery, neurology, and the pain clinic visits were reviewed.

Secondary and tertiary procedures were performed based on clinical symptoms when the response to the primary intervention was lost and symptoms recurred in a severe, medically resistant form.

### 2.1 Radiofrequency Thermocoagulation

RFTC was performed by specialized neurosurgeons. Needle insertion was carried out under local anesthesia. The puncture site was located at the mid‐pupillary line, 2.5 cm from the oral commissure, and 3 cm from the Zygomatic arch. Passage to the foramen ovale was anesthetized using 1.0% Lidocain cum Adrenalin. Needle placement was guided by X‐ray or cone‐beam computed tomography control. Once the needle reached the Gasserian ganglion, sensory and motor thresholds were tested, and stimulation in the pain area was confirmed. At this stage, patients were anesthetized, and thermocoagulation was performed under general anesthesia using conventional protocols. Heating time was typically 60 s, with temperatures gradually increased between 60°C and 80°C degrees. The number of cycles was 1–3 in most of the cases; 4–6 cycles were used in 15 of 140 patients. Thermocoagulation generators included Cosman RFG‐1A and Inomed NT2000iX. Needle used were 19 GA with a 7 mm tip for Cosman RFG‐1A and 20GA and tip 10 mm for Inomed NT2000iX generator.

### 2.2 Outcome Measurements

Data collected included sex, age, technical details of the procedure; used temperature, duration of the coagulation procedure, and measurement of sensory and motor threshold, treatment outcomes, duration of response, and complications. Outcome were assessed at the time of the procedure and at after 3 month follow‐up separately for each intervention.

Treatment response was categorized into four levels: excellent, good, minimal, and none. An excellent response meant the patients were pain‐free, and medication could be discontinued. A good response indicated patients had mild residual symptoms and might require low‐dose medication. A minimal response meant patients experienced some symptom relief, but medication could not be significantly reduced.

Perioperative complications were extracted from surgical reports and postoperative complications were identified from follow‐up records.

### 2.3 Statistics

Treatment outcomes were encoded as: 3 = excellent, 2 = good, 1 = minimal, and 0 = none, and treated as a continuous variable for analysis. This approach was chosen after statistical consultation, as ordinal data are challenging to analyze in related samples with more than two time points and the steps between categories were considered relatively uniform. A linear mixed‐effects model was applied with outcome score, RFTC time (primary, secondary, or tertiary), sex, and age as fixed effects, and a random intercept for subjects to account for repeated measures. An unstructured covariance structure was used.

Dichotomous data were analyzed using the Generalized Estimating Equations (GEE). A binomial logistic regression model was employed to account for the repeated measures design, with complication status (no complication or any complication), RFTC time, sex, and age as predictors. Statistical significance was set at *p* < 0.05. Analyses were performed using IBM SPSS Statistic 29.0 software.

## 3 Results

**FIGURE 1:**
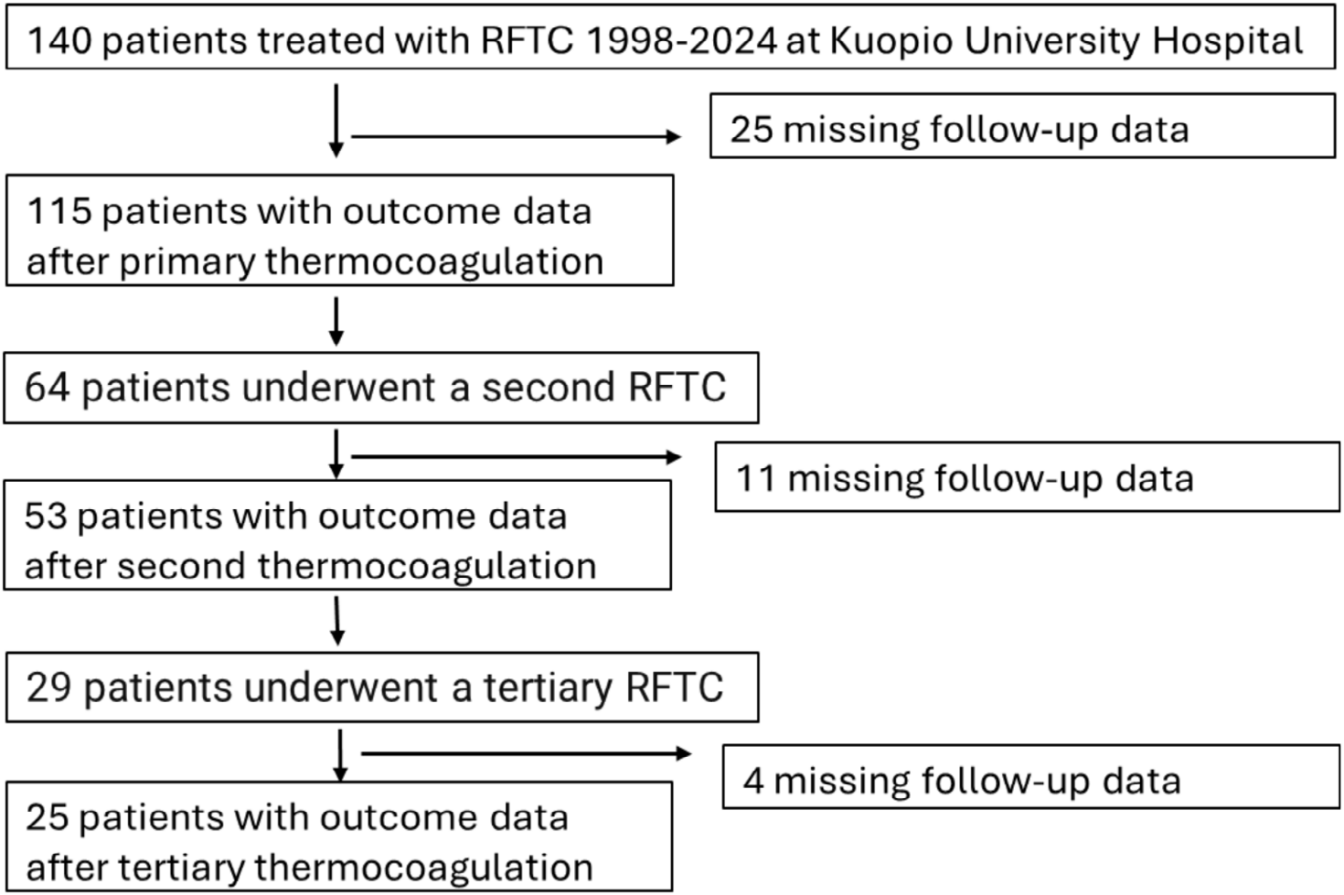
Flowchart for study population. RFTC, radiofrequency thermocoagulation.

Comprehensive medical records following the primary procedure were available for 140 patients (Figure 1, Table 1). Of these, 55% were women, and mean age at symptom onset was 66.2 years (range: 24–91 years). Treatment outcome data were available for 115 patients included in the analysis. A second RFTC was performed in 64 patients, and 29 patients underwent the third procedure.

**TABLE 1:** Characteristics for all study patients.

Total sample size, *n*	140
Sex, *n* (%)	
Male	63 (45.0)
Female	77 (55.0)
Surgical characteristics	
Primary RFTC, *n* (%)	140 (100%)
Median age (range) at RFTC in years	66.2 (24–91)
Treatment outcome available, *n* (%)	115 (82.1)
Complication after procedure, *n* (%)	22 (15.7)
PTTN, *n* (%)	2 (1.4)
Secondary RFTC, *n* (%)	64 (45.7)
Median age (range) at RFTC in years	71.9 (26–93)
Treatment outcome available, *n* (%)	53 (82.8)
Complication after procedure, *n* (%)	11 (17.2)
PTTN, *n* (%)	2 (3.1)
Tertiary RFTC, *n* (%)	29 (20.7)
Median age (range) at RFTC in years	72.8 (27–87)
Treatment outcome available, *n* (%)	25 (86.2)
Complication after procedure, *n* (%)	12 (41.4)
PTTN, *n* (%)	4 (13.8)

Abbreviations: PTTN, painful post‐traumatic trigeminal neuropathy; RFTC, radiofrequency thermocoagulation.

**FIGURE 2:**
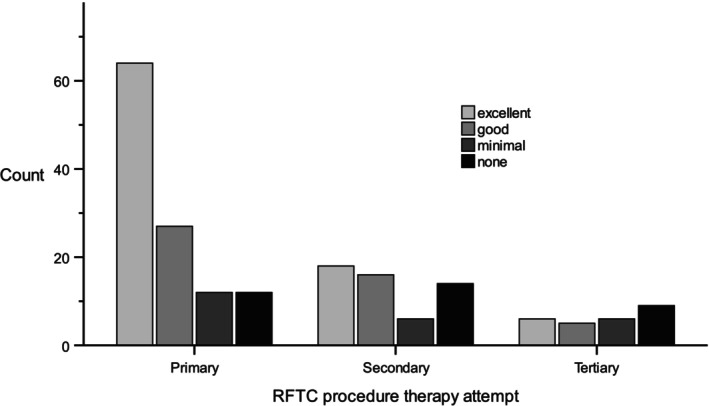
Response for RFTC after primary procedure, first re‐RFTC and second re‐RFTC, 3 months after treatment. RFTC, radiofrequency thermocoagulation.

Treatment response was assessed 3 months after RFTC (Figure 2, Table 2). In a linear‐mixed model, treatment response was significantly better after the primary procedure compared to the secondary and tertiary procedures (estimated means: primary 2.26 [95% CI: 2.08–2.45], secondary 1.69 [95% CI: 1.37–2.00], and tertiary 1.23 [95% CI: 0.78–1.70]). There was no statistically significant difference between the second and third procedure (*p* = 0.098). The proportion of patients with excellent or good outcomes was 79.2% after the primary RFTC, 62.9% after the secondary RFTC, and 42.3% after the tertiary procedure. Non‐responders increased from 10.4% after the first procedure to 25.9% and 34.6% after the second and third procedures, respectively. Patient‐reported duration of treatment response was available for 60 patients following the primary procedure. The mean duration of pain relief was 1.67 years (range: 0.1–12.0, *n* = 53) in patients without complications and 2.0 years (range: 0.5–9.5, *n* = 7) in those with complications, with no significant difference between groups (*p* = 0.402).

**TABLE 2:** Outcome of RFTC.

	Sample size, *n*	Outcome^a^, ^b^		*p* ^c^
		Estimated mean outcome (95% CI)	Estimated mean difference (95% CI)	
Primary	115	2.26 (2.08–2.45)	Reference	
Secondary	54	1.69 (1.37–2.00)	0.58 (0.23–0.93)	0.002
Tertiary	26	1.23 (0.78–1.70)	1.03 (0.51–1.55)	< 0.001

Abbreviations: CI, confidence interval; RFTC, radiofrequency thermocoagulation.

^a^ Outcome for RFTC treatment for the analysis was noted with the following values: 3 = excellent, 2 = good, 1 = minimal, and 0 = none.

^b^ Repeated‐measures linear mixed model with age at RFTC and sex were used to for estimating means and between‐group differences.

^c^ *p*‐value compares the following groups: primary and secondary, and primary and tertiary.

**FIGURE 3:**
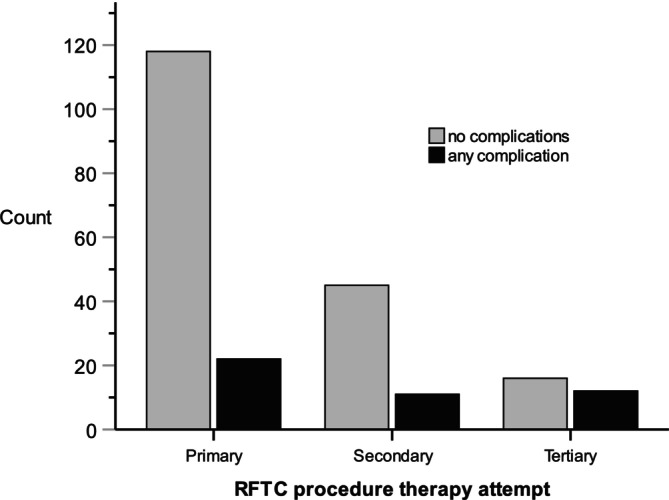
Presence of complication after primary RFTC procedure, secondary RFTC and tertiary RFTC.

**FIGURE 4:**
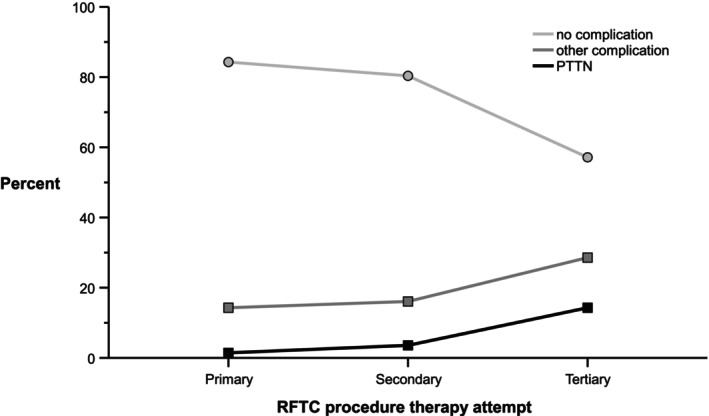
Presence of PTTN after primary RFTC procedure, secondary RFTC and tertiary RFTC. PTTN, painful post‐traumatic trigeminal neuropathy; RFTC, radiofrequency thermocoagulation.

Complication rates were 15.7% (22 patients) after the primary RFTC, 19.6% (11 patients) after the secondary RFTC and 42.9% (11 patients) after the tertiary procedure (Figure 3). In the binomial logistic regression model, female sex (OR 3.17, 95% CI: 1.47–6.85, *p* = 0.003) and tertiary procedure (OR 4.91, 95% CI: 1.93–12.5, *p* < 0.001) were significantly associated with increased complication risk (Table 3). The most severe complication, PTTN, occured in 2 patients (1.4%) after the primary procedure, 2 patients (3.1%) after the second, and 4 patients (13.8%) after the third procedure (Figure 4, Table 1).

**TABLE 3:** Risk of complication after RFTC.

	Risk of complication^a^		*p* ^b^
	OR	95% CI	
Characteristics			
Sex, female	3.17	1.47–6.85	0.003
Age	0.99	0.97–1.01	0.434
RFTC treatment			
Primary	Reference		
Secondary	1.35	0.59–3.10	0.475
Tertiary	4.91	1.93–12.5	< 0.001

Abbreviations: CI, confidence interval; RFTC, radiofrequency thermocoagulation.

^a^ Binomial logistic regression model with complication status, RFTC time, sex, and age at RFTC.

^b^ *p*‐value compares patients with complications to patients without complications after RFTC treatment, with higher OR signaling higher risk of any complication.

## 4 Discussion

According to the European Academy of Neurology (EAN) guidelines, operative treatment becomes an option when TN symptoms become chronic, medical treatment provides insufficient relief, or patients experience severe intolerance to medication. Approximately 33%–50% of TN patients ultimately required surgical intervention [2, 8]. RFTC is considered an effective and safe procedure for chronic, drug‐resistant TN in selected patients [13]. However, there is no standardized RFTC protocol, and comparative data on procedural details remain incomplete. Variations exist in puncture side, imaging guidance (fluoroscopy vs. CT‐fluoroscopy), and thermocoagulation technique (pulsed vs. continuous), as well as temperature, duration, and voltage settings [11, 13]. These differences complicate outcome comparisons and hinder the development of unified guidelines.

The concept of a “repeated procedure” also varies in the literature. In some protocols, it refers to a second intervention performed within 24–48 h during the same hospital stay, whereas in others, it denotes a procedure performed after the loss of response to the initial intervention. Few studies have examined outcomes and complication rates after repeated procedures. Liu et al. [14] compared primary and same‐hospital repeated RFTC, where the second procedure was performed 48 h after the first if no initial response occurred; no differences in response or complication rates were observed at 2‐year follow‐up. When RFTC was repeated after loss of primary response, studies reported slightly shorter response duration and fewer excellent responders after the second procedure compared to the first, but complication rates were not documented [15, 16]. In our cohort, response duration was patient reported during post‐operative visit or prior to subsequent procedures. This measure likely contains sources of error, both over‐ and underestimation; therefore, response quality was considered a more reliable metric. To our knowledge, detailed analysis of complication prevalence after repeated procedures has not been previously reported.

Our findings align with existing literature regarding response rates after primary and secondary interventions (Table 1, Figure 3). However, the third procedure and female sex were significantly associated with increased complication risk (Table 3). Common complications of ablative treatments include reduced facial sensation (19%–100%), dysesthesia (6%), reduced corneal sensation (5%), and trigeminal motor weakness such as masticatory weakness (5%) [2, 8, 9]. Sensory loss results from deliberate damage to pain‐sensing fibers and is often used as an indicator of the correct targeting, explaining reports of near‐universal short‐term sensory loss in some studies [14, 16]. Sensation typically recovers over time. Damage to the ophthalmic branch can lead to the loss of corneal sensation, predisposing to corneal ulcers, keratitis, and other infections. Corneal complications are minimized by targeting branches two and three on the Gasserian ganglion and confirming localization through sensory testing under local anesthesia prior to thermocoagulation.

The most severe complication, PTTN, is a chronic, debilitating condition following trigeminal nerve trauma. Its reported incidence ranges from 0% to 5% after ablative procedures [2, 9]. No significant differences in efficacy or safety have been demonstrated among ablative techniques [5, 9].

Risk of PTTN and other severe complications correlates with higher coagulation temperatures (> 80°C), whereas optimal outcomes with minimal complications are achieved at 60°C–75°C [17, 18]. Pulsed heating appears safer than continuous heating [17, 18]. In our study, temperatures ranged from 60°C to 80°C, with pulses under 60 s, yet PTTN still occurred (Table 4), highlighting the role of mechanical trauma. The pathophysiology of PTTN remains poorly understood; proposed mechanisms include trauma‐induced inflammation, ganglionic and central sensitization, and possible genetic preposition [12, 19]. Repeated procedures increase cumulative neural damage, further predisposing to complications [3, 19]. Based on our data, the risk associated with a third procedure outweighs potential benefits (Table 3).

**TABLE 4:** Details of patients with PTTN.

	Gender	Age	Primary procedure			Secondary procedure			Tertiary procedure	
			TC protocol	Response	Length of response (years)	TC protocol	Response	Length of response (years)	TC protocol	Response
Primary	Female	77	70°C 60 s	PTTN						
	Female	27	60°C 60 s, 65°C 60 s, 70°C 60 s	PTTN						
Secondary	Male	35	65°C 60 s	Good	1	60°C 60 s, 65°C 60 s	PTTN			
	Female	25	Uncertain needle location, no TC	None	—	70°C 60 s, 70°C 60 s	PTTN			
Tertiary	Female	52	65°C 60 s	Mild	0.75	Uncertain needle location, no TC	None	—	75°C 60 s	PTTN
	Male	78	65°C 60 s, 70°C 60 s	Good	0.2	75°C 60 s	Exellent	0.3	65°C 60 s	PTTN
	Female	64	75°C 60 s, 72°C 60 s	Exellent	1	75°C 60 s, 80°C 60 s	Mild	0.3	60°C 60 s, 65°C 60 s, 70°C 60 s, 75°C 60 s	PTTN
	Female	76	70°C 60 s, 75°C 60 s	Exellent	2	60°C 60 s, 65°C 60 s, 70°C 60 s, 75°C 60 s	Mild	1.5	60°C 60 s, 65°C 60 s, 70°C 60 s, 75°C 60 s	PTTN

All ablative techniques and stereotactic radiosurgery exhibit diminishing effectiveness over time, often necessitating additional interventions or medication. Our findings support previous observations that, response rates decline significantly after the second intervention (Figure 1). Compared to radiosurgery, ablative procedures provide more immediate and longer‐lasting pain relief [3]. Reported pain‐free rates range from 26% to 82%, for RFTC, with 55%–80% for balloon compression, and 19%–58% for chemical ablation over a 4–11 years follow‐up [9]. Loss of RFTC efficacy may reflect similar pathophysiological mechanisms underlying TN progression including demyelination, axonal dysfunction, trigeminocervical circuit alterations, and central sensitization [3, 5, 8, 20, 21]. Recent functional‐MRI studies have demonstrated changes in brain connectivity in TN patients [22, 23].

Our results also indicate a higher complication risk in women (Table 3). TN prevalence is approximately 1.5‐fold higher in women, and gender differences in TN subtypes have been reported [8]. Idiopathic TN is more common, in women, who also exhibit higher recurrence rates and poorer outcomes after microvascular decompression [24, 25]. In men, stronger vascular compression and trigeminal root atrophy are observed radiologically, and elevated blood pressure correlates with more severe findings [24].

It has been suggested that genetic factors may influence TN pathophysiology, supported by clinical observations of sex‐related differences [8]. Genetic, molecular, and Mendelian randomization studies indicate potential roles for ion channel mutations, altered ionotropic channel activity, oxidative stress pathways, inflammatory mediators, and microRNA expression [8, 26, 27, 28, 29]. Although familial clustering has been reported, environmental factors may also contribute [2, 26, 30]. The FinnGen study, a large‐scale genomic initiative based on Finnish Biobank data, provides insight into this question [31]. We reviewed the latest public release (R12) GWAS summary statistics for TN, which included 2226 diagnosed patients. No genome‐wide significant variants were identified, suggesting that TN does not have a strong monogenic basis in the Finnish population. These findings support the hypothesis that TN has a polygenic and complex genetic background, with epigenetic mechanisms and central sensitization likely playing key roles in disease progression and treatment response [21].

The retrospective design of this study introduces inherent limitations. Data were collected over an extended period, and procedures were performed by multiple surgeons, resulting in variability in technique including differences in temperature settings and heating duration. These factors may have influenced complication rates. Future research should focus on establishing standardized treatment protocols and implementing prospective registries to enable more reliable comparisons. Additionally, a deeper understanding of TN pathogenesis may open opportunities for improved treatment methods.

## 5 Conclusions

RFTC remains an effective and generally safe treatment for idiopathic TN when conservative therapy fails. The procedure can be safely repeated once. However, the risk of complications significantly increases after a third intervention, whereas clinical benefit declines. Serious complications, including PTTN, become more frequent with repeated procedures. Based on these findings, the benefit–risk ratio does not justify a tertiary RFTC in most cases, and reintervention should be considered carefully.

## Author Contributions

Data collection: S.M. and H.‐K.J.; data analyses and statistics: A.L. and H.‐K.J.; FinnGen data analyses: A.L. and S.H.; S.M. and H.‐K.J. main contribution on writing of manuscript; J.H., V.L., T.K., and K.L. contribution on design of research and in the examination of conclusions. All authors read and approved the final manuscript.

## Funding

The authors have nothing to report.

## Ethics Statement

This study is part of a broader research project on pain neuromodulation treatments (SCS Pain) and has received approval from the Ethics Committee of Kuopio University Hospital. The research permit number is ETMK 27/2014 with amendment 169/2016.

## Consent

The authors have nothing to report.

## Conflicts of Interest

The authors declare no conflicts of interest.

## Data Availability

The data that support the findings of this study are available on request from the corresponding author. The data are not publicly available due to privacy or ethical restrictions.
